# Risk Stratification of Differentiated Thyroid Cancer at King Abdullah Specialized Children's Hospital Endocrinology Clinic in Riyadh, Saudi Arabia

**DOI:** 10.7759/cureus.51372

**Published:** 2023-12-30

**Authors:** Raed Al-Dahash, Abdullah Alsohaim, Ziyad N Almutairi, Khaled Z Almutairi, Abdulkarim Alharbi, Sulaiman Alayed, Abdullah Almuhanna, Rayan Alotaibi

**Affiliations:** 1 Medicine, King Abdulaziz Medical City, National Guard, Riyadh, SAU; 2 Medicine/Endocrinology, King Abdulaziz Medical City, Riyadh, SAU; 3 Internal Medicine, King Saud Bin Abdulaziz University for Health Sciences, Riyadh, SAU; 4 College of Medicine, King Saud Bin Abdulaziz University for Health Sciences, Riyadh, SAU

**Keywords:** thyroid cancer, prevalence, ptc, recurrence, risk stratification

## Abstract

Background

Papillary thyroid cancer (PTC), a well-differentiated form of cancer, accounts for the majority of thyroid malignancies, and the incidence of PTC is on the rise. While the rate of PTC recurrence is considered to be low, there are broad spectrums of clinical and biological behavior that can lead to disease recurrence. The American Thyroid Association (ATA) risk stratification system for differentiated thyroid cancer is used as a prognostic tool to guide decision-making and management strategies most likely to achieve a favorable outcome.

Aim

This study aimed to estimate the prevalence of PTC recurrence in each category of the ATA risk stratification system and determine the appropriate iodine dose to be administered at the King Abdulaziz Medical City Endocrinology Clinic in Riyadh, Saudi Arabia.

Methods

A cross-sectional retrospective chart review was conducted on adult patients with PTC who underwent thyroidectomy procedures at the King Abdullah Specialized Children's Hospital (KASCH) Endocrinology Clinic in Riyadh between 2015 and 2023. IBM SPSS (Statistical Package for the Social Sciences) version 25 (IBM Corp., Armonk, NY) was used for data analysis.

Results

Of the 697 patients included in the study, 82.4% were females. About 5% had suffered from PTC recurrence, and 54.4% had low-risk stratification. In addition, more than half (52.1%) had received radioactive iodine (RAI). The recurrence of PTC was significantly associated with age (P = 0.019), ATA risk stratification (P = 0.0001), RAI therapy (P = 0.001), and iodine dosage (P = 0.013).

Conclusion

Both low PTC recurrence rates and low-risk stratification were observed among the PTC patients. The risk factors relating to PTC recurrence included high-risk stratification, advanced age, RAI therapy, and the dosage of RAI administered.

## Introduction

Thyroid tumors are classified as follicle-derived neoplasms (thyroid epithelial), non-epithelial tumors, other epithelial tumors, and secondary tumors based on genetic, pathological, and clinical characteristics [1,2]. In Saudi Arabia, the incidence of thyroid cancer has increased at an alarming rate of between 15% and 30% [3].

Papillary thyroid cancer (PTC) is the most common form of well-differentiated thyroid cancer (TC) and the most prevalent type found in Saudi Arabia [3]. Accounting for almost 89.4% of all thyroid malignancies, it represents the primary histology identified in patients exposed to radiation [4]. Current statistics indicate that almost 1.3% of males and females will be diagnosed with thyroid cancer at some point during their lifetime [4].

While occurrences of PTC are on the rise [5], the reason for this increase is unclear, though the figures may be attributable to improvements in the diagnosis and detection of the disease [6]. The mean age of patients diagnosed with PTC is between 30 and 40 years, with women being more commonly affected than men by a ratio of 2:1 [7,8]. In Saudi Arabia, a male-to-female ratio of 24:100 was recorded in 2007 by the Saudi Registrar of Oncology [3].

The thyroid gland is highly sensitive to the influence of ionizing radiation, with both clinical and accidental exposure being linked to an increased risk of thyroid cancer [9,10]. Furthermore, there is documentary evidence to suggest that patients with PTC who were exposed to radiation from the Chornobyl accident were easily distinguishable from those with sporadic PTC and no history of radiation exposure [11].

PTC tends to follow an indolent clinical course characterized by low mortality and morbidity. It, however, presents a broad range of clinical and biological behaviors that can cause disease recurrence and mortality based on the characteristics of the tumor and patient as well as the initial management approach taken [4]. While recurrence of PTC was previously thought to be low, with 99% survival at 20 years after surgery [12], a long-term follow-up study conducted on 269 patients detected a recurrence rate of 25% over 27 years [13].

The dynamic risk stratification assessment model allows for the integration of response to therapy with the underlying biology of the individual tumor, which makes possible real-time risk assessment at any point in the course of the disease [14]. Risk stratification for differentiated thyroid cancer represents a prognostic tool, offering guidance on decisions related to the appropriate management of the disease to achieve the most favorable outcome [15]. In addition, the American Thyroid Association (ATA) modified stratification system is designed to predict the risk of persistence or recurrence of the disease more accurately [16]. It is important to note, at this stage, that highly sensitive disease-detection tools can often detect small PTC foci that may not require immediate diagnosis and management [16]. This study aimed to estimate the prevalence of PTC recurrence in each category of the ATA risk stratification system and determine the appropriate iodine dose to be administered at the King Abdulaziz Medical City Endocrinology Clinic in Riyadh, Saudi Arabia.

## Materials and methods

Study design and setting

This cross-sectional retrospective chart review was conducted at the King Abdulaziz Medical City Endocrinology Clinic in Riyadh between 2015 and 2023.

Study subjects

The study was conducted on adult patients with PTC who had undergone thyroidectomy procedures; pediatric patients and patients with other forms of thyroid cancer were excluded.

Data collection methods, instruments used, and measurements

The researchers collected the data from medical records obtained via the BESTCare system and entered it into an Excel spreadsheet. The relevant variables included age, gender, ATA risk of recurrence (low/intermediate/high), radioactive iodine (RAI) therapy administered (yes/no), and RAI dosage. The outcome variables were ATA risk of recurrence (low/intermediate/high) and radioiodine dosage, and the grouping variables were age and gender. Approval for the study was obtained from the King Abdullah International Medical Research Center (KAIMRC).

Statistical analysis

Statistical Package for Social Sciences (SPSS) version 25 (IBM Corp., Armonk, NY) was used for the data analysis. Frequency and percentage were used for the categorical data therapy, while mean values and standard deviation were used for the numerical data. Chi-square tests and t-tests were used as appropriate for each type of data, and tests were considered significant when the P-value was <0.05.

## Results

A total of 697 patients enrolled in the study; the characteristics of the patients are summarized in Table 1. The dominant age group included patients aged 50-59 years (181; 26%), while the most common age at the time of diagnosis was 40-49 years (175; 25.1%), followed by those aged 50-59 years (174; 25%). Females represented the majority of the sample (574; 82.4%). Recurrence of PTC was recorded in 35 patients (5%), whereas the remaining 662 (95%) experienced no recurrence. ATA risk stratification revealed more than half the patients (379; 54.4%) to be at low risk, while 226 (32.4%) and 92 (13.2%) were identified as being at intermediate and high risk, respectively. RAI was given to 363 patients (52.1%), with the most common dose of 100-150 being given to 145 (39.9%).

**Table 1:** Characteristics of patients

Variables	Description (n = 697)
Age (years)	
20-29	42 (6)
30-39	103 (14.8)
40-49	174 (25)
50-59	181 (26)
60-69	132 (18.9)
70 or more	65 (9.3)
Age when diagnosed (years)	
20-29	69 (9.9)
30-39	127 (18.2)
40-49	175 (25.1)
50-59	174 (25)
60-69	104 (14.9)
70 or more	48 (6.9)
Gender	
Male	123 (17.6)
Female	574 (82.4)
Nationality	
Saudi	649 (93.1)
Non-Saudi	48 (6.9)
Recurrence of papillary thyroid cancer	
Yes	35 (5)
No	662 (95)
ATA risk stratification	
Low	379 (54.4)
Intermediate	226 (32.4)
High	92 (13.2)
Radioactive iodine	
Yes	363 (52.1)
No	334 (47.9)
Radioactive iodine dose	
<50	34 (9.4)
50-100	124 (34.2)
100-150	145 (39.9)
>150	52 (14.3)
Unknown	8 (2.2)

The correlations between PTC recurrence and other variables can be seen in the recurrence rate columns in Table 2. Recurrence of PTC is significantly associated with higher age of those aged 50-69 years old (P = 0.019), those who were diagnosed at the same age of 50-69 years (P = 0.007), those with high-risk stratification (P = 0.0001), patients who received RAI (P = 0.001), and especially those who received high doses of iodine (P = 0.013).

**Table 2:** Correlation between papillary thyroid cancer recurrence and demographics (based on column percentages) PTC: Papillary thyroid cancer.

	Recurrence of PTC		
	Yes	No	
Age (years)			P-value
20-29	1 (2.4)	41 (97.6)	0.019
30-39	3 (2.9)	100 (97.1)	
40-49	2 (1.1)	172 (98.9)	
50-59	13 (7.2)	168 (92.8)	
60-69	12 (9.1)	120 (90.9)	
70 or more	4 (6.2)	61 (93.8)	
Age when diagnosed (years)			
20-29	1 (1.4)	68 (98.6)	0.007
30-39	4 (3.1)	123 (96.9)	
40-49	3 (1.7)	172 (98.3)	
50-59	16 (9.2)	158 (90.8)	
60-69	9 (8.7)	95 (91.3)	
70 or more	2 (4.2)	46 (95.8)	
Gender			
Male	7 (5.7)	116 (94.3)	0.708
Female	28 (4.9)	546 (95.1)	
Nationality			
Saudi	33 (5.1)	616 (94.9)	1.000
Non-Saudi	2 (4.2)	46 (95.8)	
ATA risk stratification			
Low	6 (1.6)	373 (98.4)	0.000
Intermediate	10 (4.4)	216 (95.6)	
High	19 (20.7)	73 (79.3)	
Radioactive iodine			
Yes	28 (7.7)	335 (92.3)	0.001
No	7 (2.1)	327 (97.9)	
Radioactive iodine dose			
<50	2 (5.9)	32 (94.1)	0.013
50-100	5 (4)	119 (96)	
100-150	10 (6.9)	135 (93.1)	
>150	10 (19.2)	42 (80.8)	
Unknown	1 (12.5)	7 (87.5)	

The correlations between ATA risk stratification and other variables are illustrated in Table 3. Significant correlations can be seen between ATA risk stratification and both the primary age groups and patients’ age at diagnosis: The most populous age range (40-59 years) indicated significantly low risk (P = 0.004) as did the most populous diagnosis-age group (40-49 years; P = 0.014). A further significant correlation was identified between ATA risk stratification and gender (P = 0.045). Notably, the highest frequency among patients reporting no recurrence of PTC tended to be in the low ATA risk category (P = 0.0001). RAI therapy was significantly associated with risk stratification (P = 0.0001), with the highest proportion of those treated with RAI tending to be in the intermediate risk category. Finally, RAI doses of 50-100 and 100-150 were associated with intermediate risk (P = 0.0001).

**Table 3:** Correlation between papillary thyroid cancer recurrence and demographics (based on row percentages)

	American Thyroid Association risk stratification			
	Low	Intermediate	High	P-value
Age (years)				
20-29	14 (33.3)	20 (47.6)	8 (19)	0.004
30-39	62 (60.2)	31 (30.1)	10 (9.7)	
40-49	109 (62.6)	50 (28.7)	15 (8.6)	
50-59	102 (56.4)	58 (32)	21 (11.6)	
60-69	62 (47)	48 (36.4)	22 (16.7)	
70 or more	30 (46.2)	19 (29.2)	16 (24.6)	
Age when diagnosed (years)				
20-29	30 (43.5)	27 (39.1)	12 (17.4)	0.014
30-39	78 (61.4)	39 (30.7)	10 (7.9)	
40-49	106 (60.6)	54 (30.9)	15 (8.6)	
50-59	96 (55.2)	53 (30.5)	25 (14.4)	
60-69	46 (44.2)	40 (38.5)	18 (17.3)	
70 or more	23 (47.9)	13 (27.1)	12 (25)	
Gender				
Male	55 (44.7)	46 (37.4)	22 (17.9)	0.045
Female	324 (56.4)	180 (31.4)	70 (12.2)	
Nationality				
Saudi	356 (54.9)	206 (31.7)	87 (13.4)	0.358
Non-Saudi	23 (47.9)	20 (41.7)	5 (10.4)	
Recurrence of PTC				
Yes	6 (17.1)	10 (28.6)	19 (54.3)	0.000
No	373 (56.3)	216 (32.6)	73 (11)	
Radioactive iodine				
Yes	89 (24.5)	190 (52.3)	84 (23.1)	0.000
No	290 (86.8)	36 (10.8)	8 (2.4)	
Radioactive iodine dose				
<50	21 (61.8)	9 (26.5)	4 (11.8)	0.000
50-100	41 (33.1)	79 (63.7)	4 (3.2)	
100-150	23 (15.9)	87 (60)	35 (24.1)	
>150	2 (3.8)	11 (21.2)	39 (75)	
Unknown	2 (25)	4 (50)	2 (25)	

## Discussion

Both PTC and follicular thyroid carcinoma are well-differentiated types of thyroid cancer, with PTC being more prevalent than follicular carcinoma. With early diagnosis, they tend to be associated with both a positive prognosis and a high survival rate [17,18]. The risk of PTC recurrence was reported to be low, with 99% survival at 20 years following surgery [12]. Based on the definition, locoregional recurrence of PTC has been identified as ranging between 1.2% and 28% [19,20]. In the current study, the PTC recurrence rate was relatively low (5%).

An earlier study of 269 patients revealed a high recurrence rate of 25%. In 11% of the cases, however, PTC recurrence occurred after more than 20 years post-treatment, which highlights the importance of life-long follow-up care for PTC patients [13]. Since the duration of patient follow-up care was not considered as part of the study, more investigation is required in this area. A higher rate of recurrence (35.17%) was reported among PTC patients from the Philippines [21].

The risk factors associated with PTC recurrence included follicular variants of PTC, advanced age, cervical lymph node involvement, and stage four tumors [22]. While our study indicated an association between recurrence and advanced age, the rate of recurrence was highest in the 50-59 and 60-69 age groups, with the rate declining among those aged 70 years and above.

There are several well-established risk factors associated with recurrence, including age ranges over 45 years, male patients, family history of PTC, and tumors greater than 4 cm in size [21]. Our study corroborated the risk factor associated with advanced age (45+), with a substantial majority of recurrent cases occurring among patients aged 50-69. However, in contrast with the earlier study, no significant association was identified between PTC recurrence rates and gender (P = 0.7). Additionally, the current study did not investigate family history or tumor sizes.

One study, which enrolled 2,538 consecutive patients treated for papillary thyroid microcarcinoma, indicated that the age range below 55 years constituted an independent risk factor for recurrence, with a hazard ratio of 2.54 (P < 0.049) [23]. This was in contrast to our study, where patients aged 50 years and above were associated with higher rates of recurrence. However, our study did not assess the hazard ratio relating to age and did not carry out a logistic regression analysis.

Other studies have demonstrated that while the female gender has a higher PTC incidence rate and the male gender is associated with a higher prevalence of advanced-stage disease, gender is not considered a risk factor for PTC recurrence [24-26]. This is in agreement with our study, in which the recurrence rate did not differ between males and females, with no significant association being found (P = 0.7).

Among PTC patients, it has been reported that those stratified as low risk account for 80% of the cases [6]. In this study, the highest proportion of patients (>50%) were classified as low risk, followed by those considered to be at intermediate risk (32.4%), while those classified as high risk represented 13.2%.

A single-center study of 340 papillary thyroid microcarcinoma patients suggested that the proportions classified as low, intermediate, and high risk were 76.77%, 16.76%, and 6.47%, respectively [15]. While there is some discrepancy in the figures relating to low, intermediate, and high risk between the previous study [15] and our own, there is an agreement regarding the dominancy of those categorized as low risk, followed by those considered to be at intermediate risk.

A further study found that the risk of recurrence in PTC patients classified as low-to-intermediate risk increased if their initial post-ablative thyroglobin level in serum was 0.3 ng/ml or higher or if they tested positive for anti-thyroglobulin antibodies [27]. While thyroglobulin levels and the detection of anti-thyroglobulin antibodies were not reported in our study, we found a significant association between risk stratification and recurrence, in that those classified as high risk were significantly susceptible to recurrence followed by those in the intermediate-risk group.

It has been noted that there is no international data on the factors affecting the recurrence of disease among low-risk PTC patients [21]. This is in agreement with our study as we were unable to determine the risk factors associated with recurrence among those classified as low, intermediate, or even high risk, owing to a very low recurrence rate (5%).

A previous study from the Philippines has revealed, via the multivariate logistic regression analysis of risk factors for recurrence among low-risk PTC patients, that tumors of diameter 2-4 cm (OR, 9.17) or >4 cm (OR, 16.46) and a family history of PTC (OR, 67.27) were significant predictors of recurrence. However, it should be noted that as the recurrence rate for the study was high (35.17%), it was possible to identify a relationship between recurrence-related risk factors and risk stratification [21]. 

The management of PTC in patients involves thyroidectomy procedures either with or without RAI therapy [21]. The thyroid gland is, however, highly sensitive to ionizing radiation, even in a clinical form [4]. Moreover, it has been observed that PTC patients who have been exposed to radiation can be easily distinguished from those with no history of radiation exposure and sporadic PTC [11], and individuals receiving radiotherapy for certain types of neck and head cancer may be at greater risk of developing thyroid cancer [9].

This study, in which more than one-half of patients had received RAI, indicated a correlation between non-recurrence and patients receiving RAI therapy, which is in agreement with the findings of previous studies. An earlier Filipino study reported that the recurrence of PTC was significantly higher among those who did not receive RAI (83.02%) compared to those who received RAI (7.61%) (P < 0.005) [21]. A study of 340 patients with papillary thyroid microcarcinoma reported that 63.53% of patients received RAI postoperatively, and of those not treated with RAI, 46.74% were classified as low risk, while 3.51% were classified as intermediate risk [15].

This study identifies significant correlations between ATA risk stratification and a range of factors including current age, age at the time of diagnosis, gender, recurrence of PTC, RAI therapy, and the dosage of RAI administered. A previous study found that the rate of recurrence was significantly higher among those with intermediate risk rather than low-risk PTC (P = 0.0005) [28]. In our study, the highest proportion of those who experienced recurrence were classified as high risk, followed by those in the intermediate risk category. However, recurrence occurred in a significant proportion of those classified as intermediate risk compared to those classified as low risk.

There are some limitations associated with our research. The study did not, for example, obtain either family histories or data relating to tumor sizes (particularly those > 4 cm), both of which have been associated elsewhere with an increased risk of recurrence [21]. Another limitation relates to the measurement of thyroglobulin levels and the detection of anti-thyroglobulin antibodies. One study has suggested that low-to-intermediate-risk PTC patients were at an increased risk of recurrence if their initial post-ablative thyroglobulin level in serum was 0.3 mg/ml or higher or if they tested positive for anti-thyroglobulin antibodies [27]. A final limitation was that we were unable to determine the risk factors relating to recurrence among those with low, intermediate, or even high risk owing to very low recurrence rates (5%).

## Conclusions

This study conducted at the King Abdulaziz Medical City Endocrinology Clinic in Riyadh, Saudi Arabia, provides critical insights into the patterns of PTC recurrence. It reveals a clear association between recurrence rates and ATA risk stratification categories, with a significant number of patients falling into the low-risk category. The study highlights that recurrence is influenced by ATA risk, age, age at diagnosis, and RAI therapy, suggesting that these factors are critical in predicting and managing PTC recurrence. Notably, our findings challenge the conventional approach of uniform RAI therapy, advocating for personalized RAI dosage based on individual patient profiles, especially considering age and therapeutic history. This approach could enhance treatment efficacy and reduce recurrence, moving beyond the one-size-fits-all strategy. This study contributes significantly to the evolving landscape of PTC treatment, emphasizing the need for personalized, patient-centric therapeutic strategies.
